# Computational Analysis Reveals the Characteristics of Immune Cells in Glomerular and Tubulointerstitial Compartments in IgA Nephropathy Patients

**DOI:** 10.3389/fgene.2022.838863

**Published:** 2022-05-04

**Authors:** Bin Li, Suchun Li, Yuting Fan, Hui Diao, Siyang Ye, Huajing Peng, Wei Chen

**Affiliations:** ^1^ Department of Nephrology, The First Affiliated Hospital, Sun Yat-sen University, Guangzhou, China; ^2^ NHC Key Laboratory of Clinical Nephrology (Sun Yat-Sen University), Guangdong Provincial Key Laboratory of Nephrology, Guangzhou, China

**Keywords:** IgA nephropathy, glomerulonephritis, immune cell landscape, macrophages, CIBERSORT, bioinformatic analysis

## Abstract

**Objective:** The commonalities and differences regarding immune states between glomerular and tubulointerstitial compartments of IgA nephropathy (IgAN) remains largely undetermined. We aim to perform bioinformatic analysis for providing a comprehensive insight into the characteristics of immune cells and associated molecular mechanisms in IgAN.

**Materials and Methods:** We performed integrated bioinformatic analyses by using IgAN-related datasets from the Gene Expression Omnibus database. First, the differentially expressed genes (DEGs) were identified and subjected to Gene Ontology (GO) and Kyoto Encyclopedia of Genes and Genomes (KEGG) pathway enrichment analyses. Then, CIBERSORT was employed to determine the landscape of infiltrating immune cells in both glomerular and tubulointerstitial compartments of IgAN patients, followed by Pearson’s correlation analysis and principal component analysis (PCA). Finally, commonly shared DEGs between glomerular and tubulointerstitial entities were recognized, followed by correlation analyses to identify the dominant commonly shared DEGs associated with immune cell infiltration in IgAN.

**Results:** GO and KEGG enrichment analyses showed apparently distinct biological processes in the glomerular and tubulointerstitial compartments of IgAN. In addition, CIBERSORT analyses revealed a clear trend of increasing proportions of M1 macrophage and M2 macrophage in the glomerular compartment while noticeably higher proportions of resting CD4^+^ memory T cells and M2 macrophages in the tubulointerstitial compartments. The PCA analyses showed that the varying composition of immune cells in both glomerular and tubulointerstitial entities was compelling to distinguish IgAN patients from healthy living controls. In addition, 21 commonly shared DEGs between glomerular and tubulointerstitial entities were recognized as key regulators in the pathogenesis of IgAN, among which the enhanced hemoglobin subunit beta (*HBB*) gene expression was found to be positively associated with M2 macrophage in the glomerular compartment and resting CD4^+^ memory T cells in the tubulointerstitial compartment. Most importantly, FBJ murine osteosarcoma viral oncogene homolog B (*FOSB*) gene deficiency was recognized as the dominant alteration in promoting M2 macrophage infiltration in the glomerular compartment of IgAN.

**Conclusion:** The findings from our current study for the first time reveal commonalities and differences regarding immune states between glomerular and tubulointerstitial compartments, as well as decode the essential role of M2 macrophages and associated molecular patterns within the microenvironments of IgAN.

## Introduction

IgA nephropathy (IgAN) is one of the most frequent types of primary glomerulonephritis and a principal cause of end-stage renal disease worldwide. The clinical manifestations of IgAN include repeated episodes of gross or microscopic hematuria, proteinuria, hypertension, loss of glomerular filtration function, and pathological changes featuring mesangial deposition of galactose-deficient IgA1 (Gd-IgA1)-containing immune complexes. Although 30–40% of patients develop end-stage renal disease within 20–30 years of diagnosis, current therapies such as blood pressure control or non-specific immune inflammation inhibition cannot impede the malignancy of IgAN, partly due to the incomplete understanding of the complicated pathogenesis.

Apart from the attack of Gd-IgA1 on mesangial cells, infiltration and activation of immune cells also provoke progressive renal lesions of IgAN. For instance, the cytokines derived from circulating CD4^+^ T cells in IgAN patients were able to trigger over-secretion of Gd-IgA1 by B cells (Yamada et al., 2010; Ruszkowski et al., 2019). In addition, glomerular CD68^+^ cells of monocyte–macrophage lineage were recognized as marker of endocapillary hypercellularity and indicator of chronic tubulointerstitial damage in IgAN (Soares et al., 2019). More importantly, probing the immune landscape of IgAN by single-cell transcriptomics revealed significantly higher proportions of macrophages and CD8^+^ T cells in the early stage of IgAN (Zheng et al., 2020). Accordingly, evaluating the immune cell status, as well as the altered gene expression patterns associated with immune cell infiltration, is important for yielding insights into the pathogenic process of IgAN.

The Cell-type Identification By Estimating Relative Subsets Of known RNA Transcripts (CIBERSORT) deconvolution algorithm method (https://cibersort.stanford.edu/) has been successfully developed to distinguish and assess the relative fraction of 22 immune cell phenotypes from bulk tissue gene expression profiles obtained by RNA-sequencing. This deconvolution technique can provide crucial information about the variation of immune cells that arise at different disease stages throughout the onset, development, and treatment, thus allowing earlier and more accurate diagnosis of comorbidities and prediction of therapeutic response.

In the present study, we for the first time found that M2 macrophage was apparently increased in both glomerular and tubulointerstitial compartments of IgAN by bioinformatic approaches. In addition, 21 differentially expressed genes (DEGs) (1 upregulated and 20 downregulated) were identified as the commonly shared DEGs between glomerular and tubulointerstitial compartments in IgA patients. Correlation analysis found that hemoglobin subunit beta (*HBB*) gene was positively associated with infiltrating M2 macrophage in the glomerular compartment and resting CD4^+^ memory T cells in the tubulointerstitial compartment of IgAN patients. Most importantly, FBJ murine osteosarcoma viral oncogene homolog B (*FOSB*) gene deficiency was identified as the dominant factor in promoting M2 macrophage recruitment and infiltration in the glomerular compartment of IgAN, and it may be a useful biomarker and therapeutic target in diagnosis and prognosis for IgAN patients.

## Materials and Methods

### Retrieval of Microarray Dataset Collection

**FIGURE 1:**
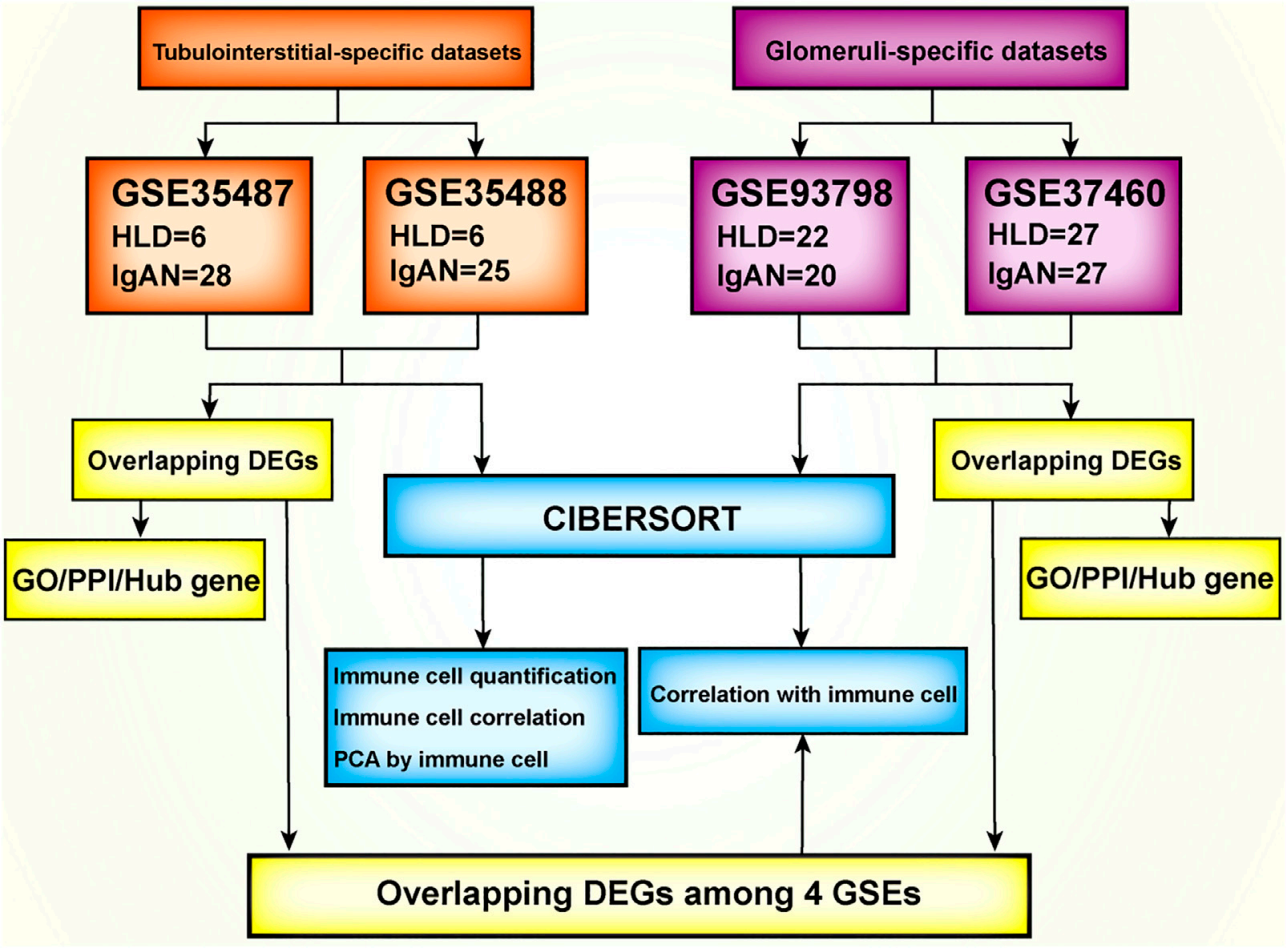
Flowchart of the analysis used in this study. GSE, gene expression data series; DEG, differentially expression genes; CIBERSORT, Cell-type Identification By Estimating Relative Subsets Of known RNA Transcripts; GO, Gene Ontology; KEGG, Kyoto Encyclopedia of Genes and Genomes; PCA, principal component analysis.

The flow diagram of the overall study design and analysis procedure is depicted in Figure 1. The qualified datasets comprised of comprehensive intrarenal gene expression profiles of kidneys from human IgAN patients were screened for further analysis. The enrollment criteria had to obey the rules as follows: 1) the study object must be human renal biopsy microarray or RNA-seq data, and samples did not pertain to different phenotypes of IgAN; 2) samples must be divided into glomeruli and tubulointerstitium from kidney biopsy samples instead of whole cortex tissue. In accordance with the aforementioned criteria, four qualified IgAN gene expression profiles (GSE93798, GSE37460, GSE35487, and GSE35488) were downloaded from the Gene Expression Omnibus (GEO) database and used to identify differentially expressed genes (DEGs). To be specific, in GSE37460 and GSE93798, RNA from the glomeruli compartment of human renal biopsy was extracted and processed for hybridization on Affymetrix microarrays, hence were exploited to profile the infiltrating immune cells in the glomerular compartment. The GSE37460 dataset included data on renal glomerular samples from patients with IgAN (*n* = 27) and healthy living donors (*n* = 27) on GPL11670 (Affymetrix Human Genome U133 Plus 2.0 Array) and GPL14663 (Affymetrix GeneChip Human Genome HG-U133A Custom CDF) platforms. GSE93798 contained data on renal glomerular samples from patients with IgAN (*n* = 20) and healthy living donors (*n* = 22) on the GPL22945 (Affymetrix Human Genome U133 Pus 2.0 Array) platform. By contrast, in GSE35487 and GSE35488, RNA from the tubulointerstitial compartment of human renal biopsy was extracted and processed for hybridization on Affymetrix microarrays, hence were employed to define the immune cell landscape in the tubulointerstitial compartment. The GSE35487 dataset included data on renal tubulointerstitial samples from patients with IgAN (*n* = 28) and healthy living donor (*n* = 6) on the GPL96 (Affymetrix Human Genome U133A Array) platform. The GSE35488 dataset included data on renal tubulointerstitial samples from patients with IgAN (*n* = 25) and healthy living donor (*n* = 6) on the GPL14663 (Affymetrix GeneChip Human Genome HG-U133A Custom CDF) platform. Additional information about all the aforementioned four datasets, including the detailed procedure of human RNA extraction from the glomeruli and tubulointerstitial compartments, sample preparation, and microarray processing as well as gene expression data analysis, could be acquired from the corresponding original literature and Supplementary Table S1 (Reich et al., 2010; Berthier et al., 2012; Liu et al., 2017).

### Identification of DEGs and Functional Enrichment Analyses

The linear models for the microarray data (LIMMA) package in Bioconductor was used to identify DEGs by comparing the expression values between kidney tissues from healthy living donor and those from IgAN patients. The corresponding *p*-values of the gene symbols after the t-test were used, and adjusted *p* < 0.05 and |log2 (FC)| > 1 were used as the selection cutoff criteria. Subsequently, the overlapping DEGs between GSE37460 and GSE93798 datasets were recognized to define glomerular compartment-specific DEGs associated with IgAN; the overlapping DEGs between GSE35487 and GSE35488 datasets were recognized to define tubulointerstitial compartment-specific DEGs associated with IgAN. These DEGs or overlapping DEGs were obtained for Gene Ontology (GO) enrichment and pathway analyses through WebGestalt (WEB-based GEne SeT AnaLysis Toolkit). In addition, the DEGs shared by both glomerular and tubulointerstitial compartments were further screened to define DEGs essential for the pathogenesis of both these two entities.

### Evaluation of Immune Cell Infiltration by CIBERSORT

The analytical platform CIBERSORT (https://cibersort.stanford.edu/) with the reference of 1000 permutations and LM22 signature was employed to characterize the infiltrating immune cell composition of kidney tissue. The CIBERSORT deconvolution algorithm has been validated to accurately and reliably calculate 22 types of immune cell fractions based on bulk transcriptome data. These immune cells are as follows: naive B cells, memory B cells, plasma cells, CD8^+^ T cells, naive CD4^+^ T cells, resting CD4^+^ memory T cells, activated memory CD4^+^ T cells, follicular helper T cells (Tfhs), regulatory T cells (Tregs), gamma delta (*γδ*) T cells, resting natural killer (NK) cells, activated NK cells, monocytes, M0 macrophages, M1 macrophages, M2 macrophages, resting dendritic cells, activated dendritic cells, resting mast cells, activated mast cells, eosinophils, and neutrophils. The notable alteration of the immune cell composition was identified according to the threshold of the Wilcoxon test with a cutoff standard p-value < 0.05. Pearson’s correlation coefficient was used to assess the relationship between different immune cell phenotypes, and principal component analysis (PCA) was utilized to determine whether there were clear boundaries in infiltrating immune cells between IgAN and healthy living donor.

### Correlation Analysis Between DEGs and Infiltrating Immune Cells

The overlapping DEGs among four datasets were further identified to define the commonly shared DEGs between glomerular and tubulointerstitial compartments, which are deemed as essential regulators associated with the pathogenesis of IgAN. Subsequently, the ggstatsplot package was used to perform correlation analysis between these commonly shared DEGs from all four datasets and infiltrating immune cells, and the ggplot2 package was used to visualize the results.

## Results

### Identification of DEGs, GO and KEGG Pathway Enrichment Analysis of DEGs

**FIGURE 2:**
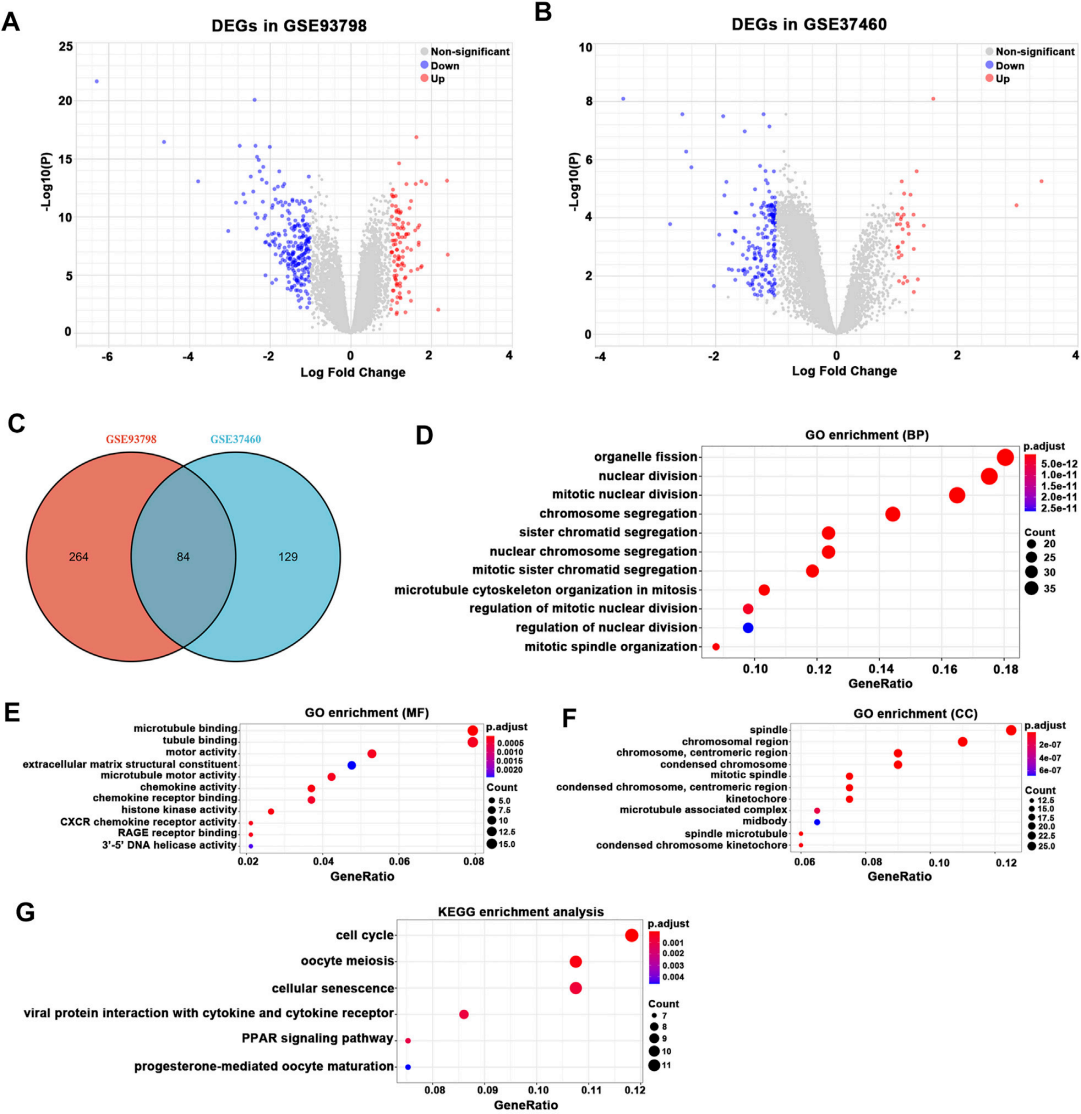
Differentially expressed gene (DEG) expression between healthy living donor and IgAN. **(A)** In total, 348 DEGs (107 upregulated and 241 downregulated) in GSE93798 were selected by volcano plot filtering (adjusted p < 0.05 and |log2 (FC)| > 1). **(B)** In total, 213 DEGs (33 upregulated and 180 downregulated) in GSE37460 were selected by volcano plot filtering (adjusted p < 0.05 and |log2 (FC)| > 1). **(C)** In total, 84 overlapping DEGs (12 upregulated and 72 downregulated) between GSE93798 and GSE37460 as showed by the Venn plot. **(D–F)** GO analyses results of DEGs. **(G)** Pathway analyses results of DEGs.

After batch correction and standardization of the microarray results, a total of 348 DEGs were detected in GSE93798 and 213 DEGs in GSE37460 (Figures 2A,B). A total of 84 overlapping DEGs between GSE93798 and GSE37460 were identified and recognized as the key DEGs associated with glomerular pathogenesis in IgAN (Figure 2C; Supplementary Table S2). Then, GO analysis and KEGG pathway analysis were performed to reveal the biological function based on the overlapping DEGs between GSE93798 and GSE37460. The top three GO terms in the biological process category were organelle fission, nuclear division, and mitotic nuclear division (Figure 2D). The top three GO terms in the molecular function category were microtubule binding, tubule binding, and motor activity (Figure 2E). The top three GO terms in the cellular component category were spindle, chromosomal region, and chromosome and centromeric region (Figure 2F). KEGG pathway analysis revealed that the overlapping DEGs were mainly enriched in cell cycle, oocyte meiosis, cellular senescence, viral protein interaction with cytokine and cytokine receptor, peroxisome proliferator-activated receptor (PPAR) signaling pathway, and progesterone-mediated oocyte maturation (Figure 2G).

**FIGURE 3:**
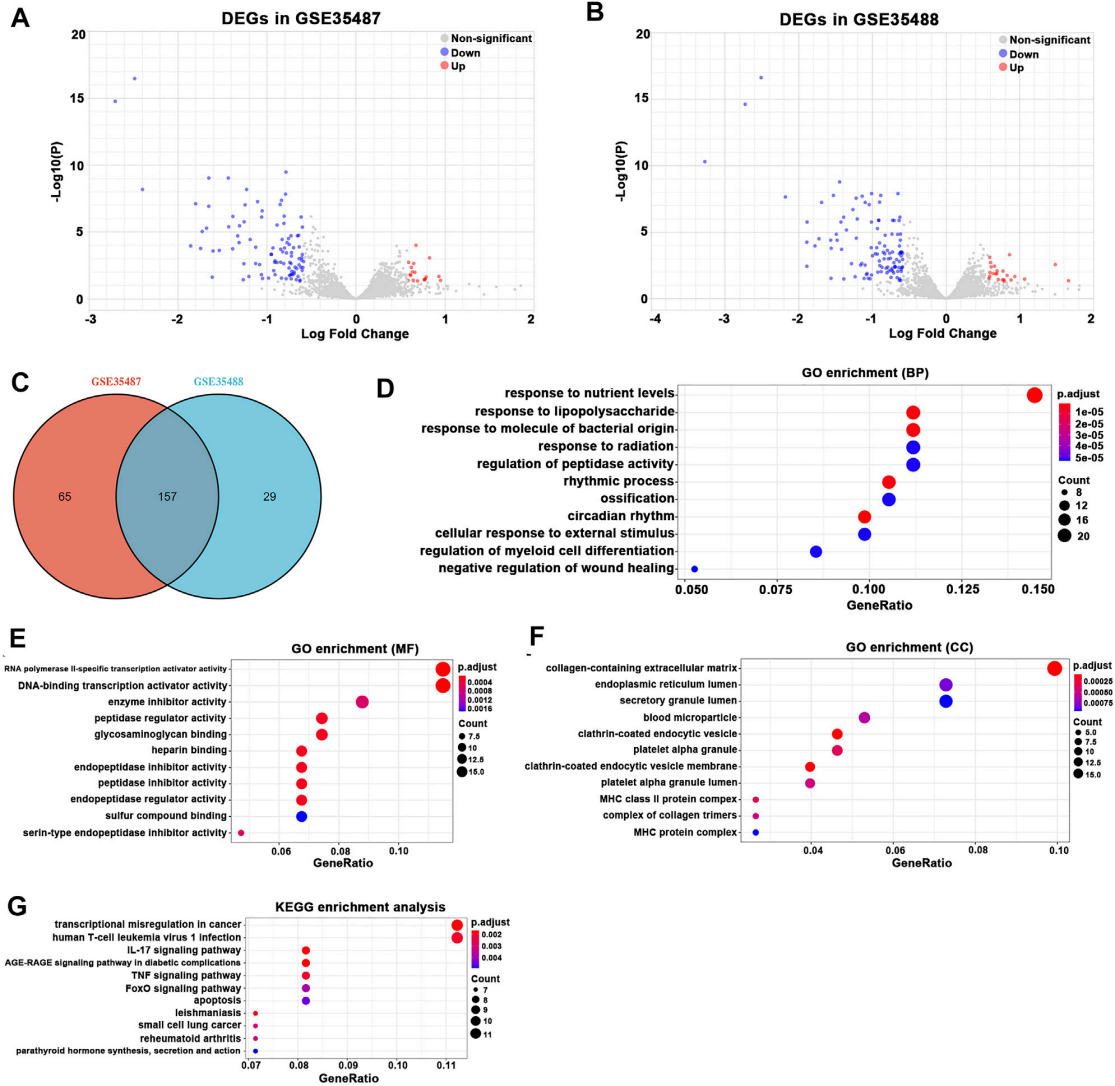
Differentially expressed gene (DEG) expression between healthy living donor and IgAN. **(A)** In total, 222 DEGs (144 upregulated and 78 downregulated) in GSE35487 were selected by volcano plot filtering (adjusted p < 0.05 and |log2 (FC)| > 1). **(B)** In total, 186 (128 upregulated and 58 downregulated) DEGs in GSE35488 were selected by volcano plot filtering (adjusted p < 0.05 and |log2 (FC)| > 1). **(C)** In total, 157 overlapping DEGs (113 upregulated and 44 downregulated) between GSE35487 and GSE35488 as showed by the Venn plot. **(D–F)** GO analyses results of DEGs. **(G)** Pathway analyses results of DEGs.

On the other hand, a total of 222 DEGs were detected in GSE35487 and 186 DEGs in GSE35488 (Figures 3A,B). A total of 157 overlapping DEGs between GSE35487 and GSE35488 were identified and recognized as the key DEGs associated with tubulointerstitial pathogenesis in IgAN (Figure 3C; Supplementary Table S3). Overlapping DEGs between GSE35487 and GSE35488 then experienced GO functional enrichment analysis, with the three most significant GO terms in the molecular function category being response to nutrient levels, response to lipopolysaccharide, and response to molecule of bacterial origin (Figure 3D), the three most significant GO terms in the molecular function category being RNA polymerase II-specific transcription activator activity, DNA-binding transcription activator activity, and enzyme inhibitor activity (Figure 3E), and the three most significant GO terms in the cellular component category being collagen-containing extracellular matrix, endoplasmic reticulum lumen, and secretory granule lumen (Figure 3F). KEGG pathway analysis revealed that the overlapping DEGs were mainly enriched in the transcription misregulation in cancer, human T-cell leukemia virus 1 infection, and interleukin-17 (IL-17) signaling pathway (Figure 3G). The difference in the GO and KEGG pathway analyses between glomerular and tubulointerstitial compartments highlighted the difference in the microenvironment between glomerular and tubulointerstitial entities during the onset and progress of IgAN.

### Composition of Immune Cell by CIBERSORT

**FIGURE 4:**
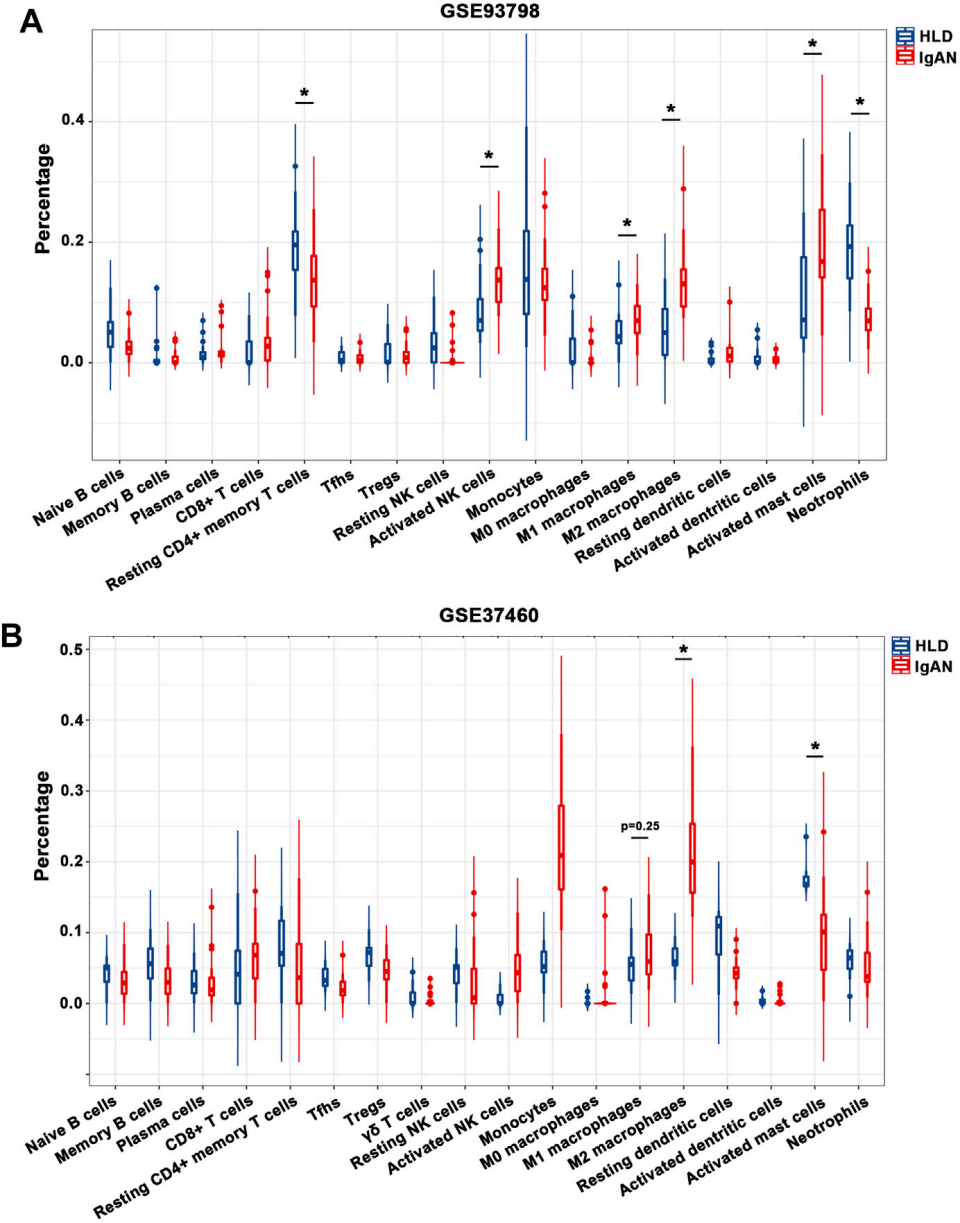
Composition of infiltrating immune cell subpopulations in kidney tissues from IgAN patients. **(A)** Violin diagram of the proportion of immune cells in GSE93798. **(B)** Violin diagram of the proportion of immune cells in GSE37460. HLD, healthy living donor; IgAN, IgA nephropathy.

**FIGURE 5:**
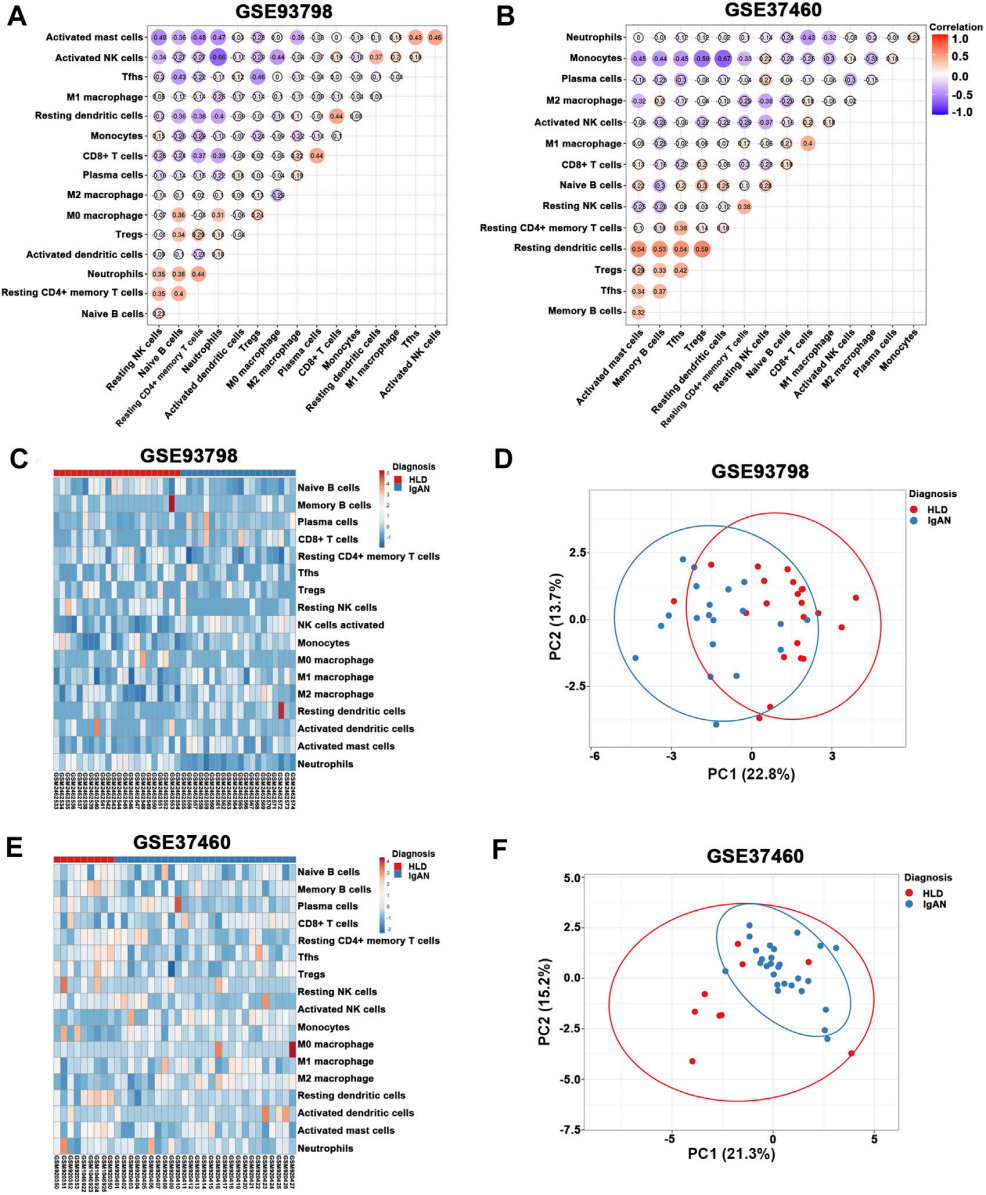
**(A,B)** Correlation heat map of 22 types of immune cells in GSE93798 and GSE37460. Dot size shows the extent of their relationships, and dot color indicates if they are positively related (red dots) or negatively related (blue dots). The number inside dots indicates the corresponding correlation value between two immune cells. The darker the color, the stronger the correlation. Colored dot without a black line around it denotes significance (*p* < 0.05). **(C,D)** Heat map and principal component analysis (PCA) cluster plot of immune cells in GSE93798. **(E,F)** Heat map and PCA cluster plot of immune cells in GSE37460.

The percentage of infiltrating immune cells based on the aforementioned RNA expression data was analyzed by CIBERSORT. Compared to healthy living control, glomerular IgAN was characterized by a clear trend of increasing M1 macrophage and M2 macrophage (Figures 4A,B; Supplementary Table S4). Particularly, M2 macrophage was dramatically elevated in both GSE93798 and GSE37460, accounting for the most pronounced elevation among all the immune cell types in glomerular IgAN (Figures 4A,B). Furthermore, a significantly negative correlation between M2 macrophage and activated mast cells was presented in both GSE93798 (*r* = −0.36) and GSE37460 (*r* = −0.32) by the correlation analyses (Figures 5A,B). A negative correlation between M2 macrophage and resting NK cells (*r* = −0.38) and a positive correlation between M1 macrophage and CD8^+^ T cell (*r* = 0.4) were presented in GSE37460 (Figure 5B). In addition, IgAN and healthy living donor display a clear separation when PCA was performed on the basis of the glomerular immune cell status, with the first two PCs explaining 36.5% of the variance in GSE93798 (Figures 5C,D) and 36.5% of variance in GSE37460 (Figures 5E,F). The first two PCs could explain most of the data variation and confirm the sensitivity and specificity of the altered immune cell status in glomeruli to discriminate healthy living donors from IgAN patients. These results underpinned the vital role of macrophages in the development of IgAN, which is in line with the classic notion that interaction between mesangial cells and immune cells accelerates the progress of IgAN.

**FIGURE 6:**
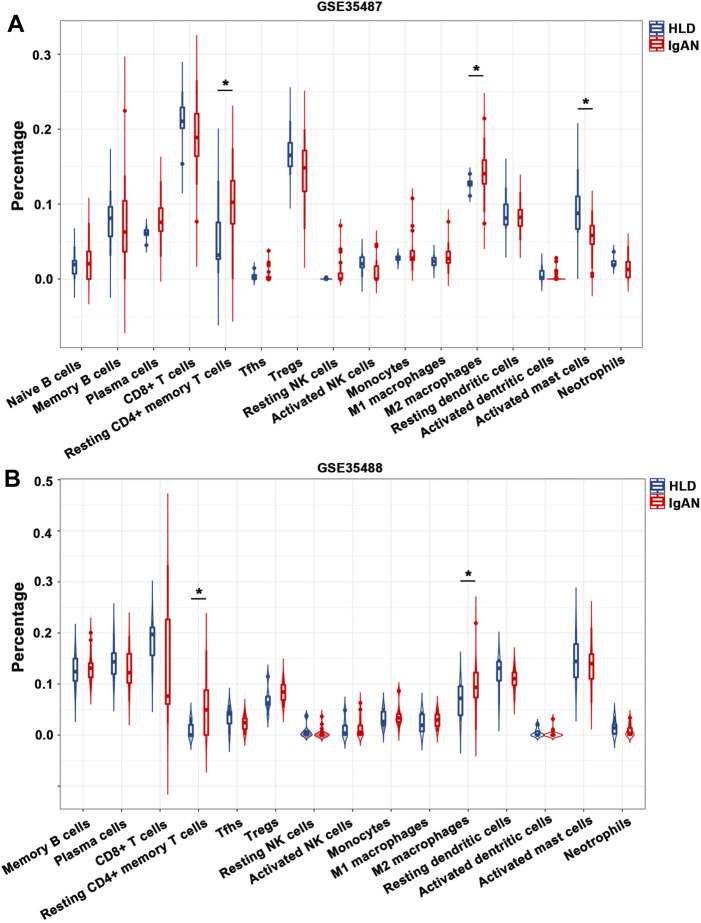
Composition of infiltrating immune cell subpopulations in kidney tissues from IgAN patients. **(A)** Violin diagram of the proportion of immune cells in GSE35487. **(B)** Violin diagram of the proportion of immune cells in GSE35488. HLD, healthy living donor; IgAN, IgA nephropathy.

**FIGURE 7:**
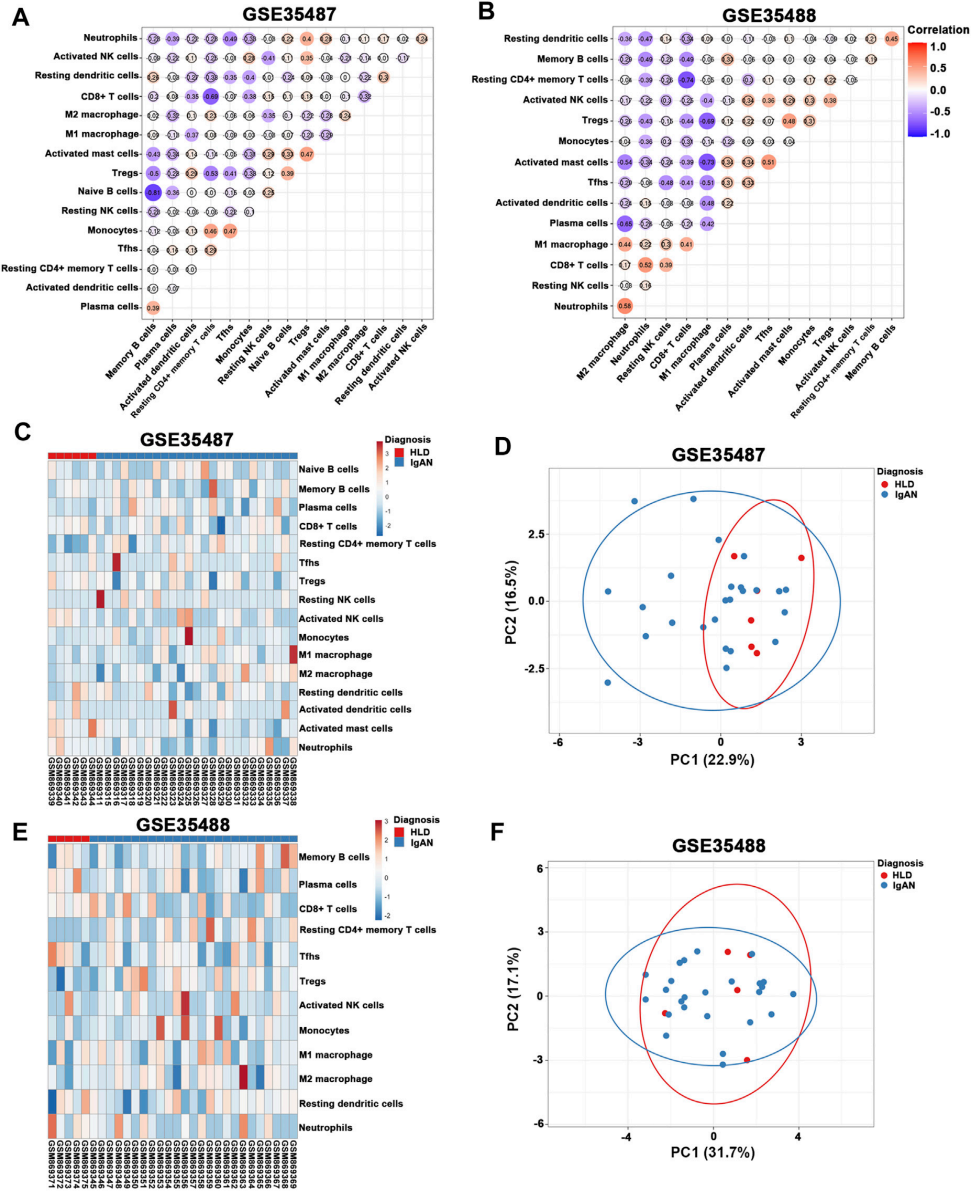
**(A,B)** Correlation heat map of 22 types of immune cells in GSE35487 and GSE35488. Dot size shows the extent of their relationships and dot color indicates if they are positively related (red dots) or negatively related (blue dots). The number inside dots indicates the corresponding correlation value between two immune cells. The darker the color, the stronger the correlation. Colored dot without a black line around it denotes significance (*p* < 0.05). **(C,D)** Heat map and principal component analysis (PCA) cluster plot of immune cells in GSE35487. **(E,F)** Heat map and PCA cluster plot of immune cells in GSE35488.

In the meantime, tubulointerstitial IgAN was featured by a noticeably higher proportions of resting CD4^+^ memory T cells and M2 macrophages in both GSE35487 and GSE35488 (Figures 6A,B; Supplementary Table S5). More importantly, correlation analysis found that the percentages of M2 macrophages was negatively correlated with resting NK cells (*r* = −0.35) in GSE35487 (Figure 7A). By comparison, the percentage of M2 macrophages was positively correlated with neutrophils (*r* = 0.58) and M1 macrophage (r = 0.44) but negatively correlated with plasma cells (*r* = −0.65), activated mast cells (*r* = −0.54), and resting dendritic cells (*r* = −0.36) in GSE35488 (Figure 7B). In addition, the percentage of resting CD4^+^ memory T cells was found negatively correlated with CD8^+^ T cells in both GSE35487 (*r* = −0.69) and GSE35488 (*r* = −0.74) (Figures 7A,B). In addition, PCA analyses demonstrated an obvious clustering trend for the immune cell status in tubulointerstitial between the healthy living donor and IgAN patient, with the first two PCs explaining 39.4% variation in GSE35487 (Figures 7C,D) and 48.8% variation in GSE35488 (Figures 7E,F), suggesting the capability of the immune cell status in tubulointerstitial to distinguish healthy living donors from IgAN patients. This finding revealed important meanings of the dysregulated immune cell status in tubulointerstitium during the development of IgAN. These observations indicate that the selective accumulation and exudation of M2 macrophages may be precursor steps in the evolution of IgAN.

### FOSB Identified as a Key Factor of Glomerular Immune Infiltration in the Microenvironment of IgA Nephropathy

Given our findings showed the crucial role of dysregulated immune cell infiltration, particularly M2 macrophages in both the glomerular and tubulointerstitial compartments of IgAN, we are very interested to know what are the commonly shared regulators that contribute to the immune cell infiltration in both these two entities. These commonly shared regulators might help shed light on the commonalities and differences between glomerular and tubulointerstitial compartments in the development of IgAN.

**FIGURE 8:**
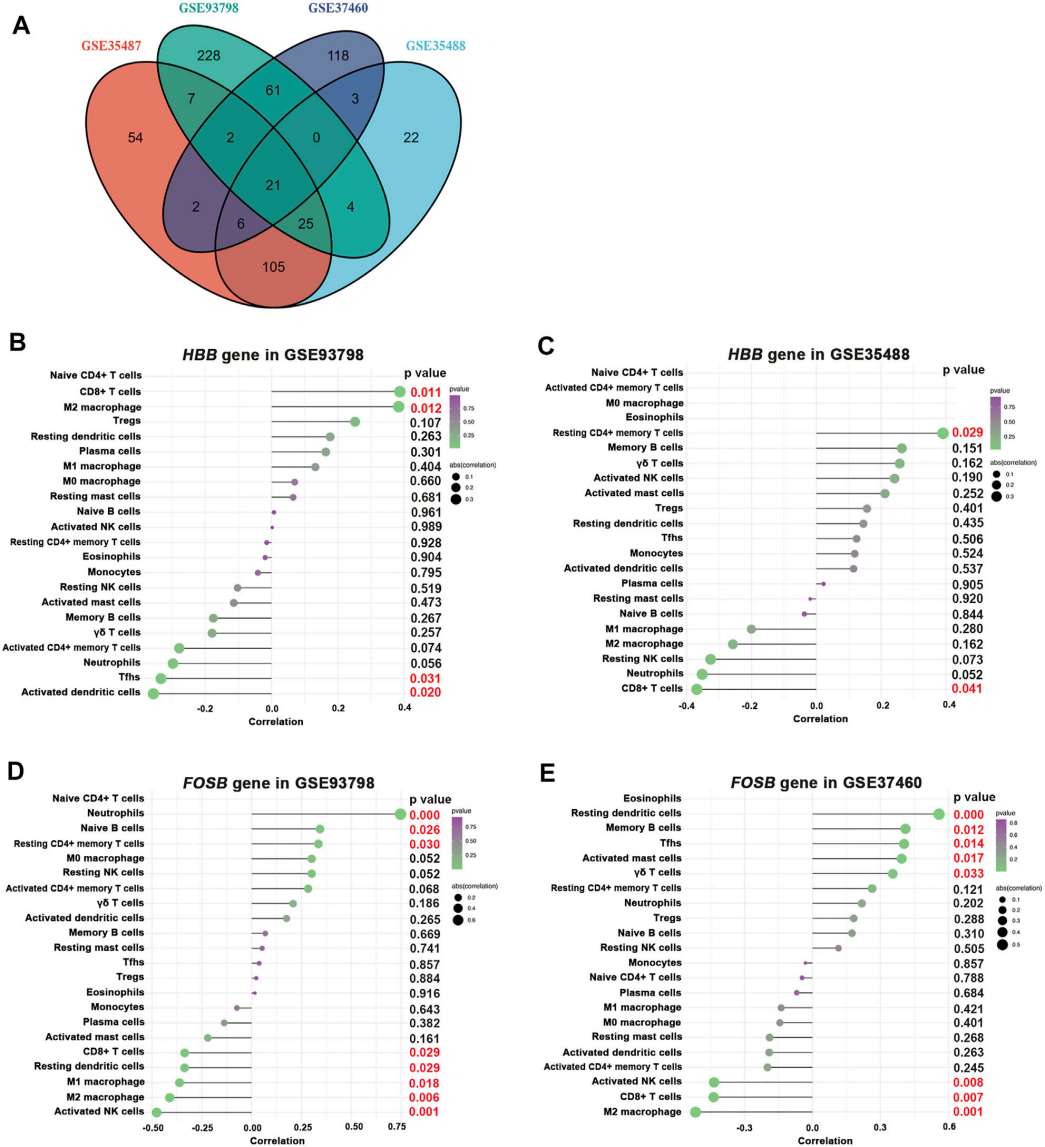
Correlation between *HBB*, *FOSB* gene, and infiltrating immune cells. **(A)** Overlapping DEGs among all selected four GEO datasets. **(B,C)** Correlation between *HBB* gene and infiltrating immune cells in GSE93798 and GSE35488, respectively. **(D,E)** Correlation between *FOSB* gene and infiltrating immune cells in GSE83798 and GSE37460, respectively.

The results revealed that 21 DEGs were shared exclusively by glomerular and tubulointerstitial compartments (Figure 8A and Table 1), including 1 upregulated gene (HBB) and 20 downregulated genes. Intriguingly, as the only upregulated DEGs in the 21 overlapping DEGs among all four datasets, *HBB* was positively associated with CD8^+^ T cells in the glomerular compartment of GSE93798 (Figure 8B), but negatively associated with CD8^+^ T cells in the tubulointerstitial compartment of GSE35488 (Figure 8C), indicating the heterogeneity between tubulointerstitial and glomerular compartments in initiating the immune infiltration in IgAN. In addition, *HBB* was positively associated with M2 macrophage in the glomerular compartment of GSE93798 (Figure 8B) as well as resting CD4^+^ memory T cells in the tubulointerstitial compartment of GSE35488 (Figure 8C). Most importantly, as the dominant downregulated DEGs in the glomerular compartment among the 21 overlapping DEGs, *FOSB* gene was found to be negatively correlated with M2 macrophages, CD8^+^ T cells, and activated NK cells in both glomerular datasets (GSE93798 and GSE37460), indicating the key role of *FOSB* deficiency in the glomerular compartment of IgAN as the driving molecular force during the process of immune cell activation (Figures 8D,E). Special noteworthy should be paid to the obviously negative correlation between *FOSB* gene and M2 macrophage, considering this result is consistent with the aforementioned data recognizing M2 macrophage as the most pronounced elevation among all the immune cell types in the glomerular compartment of IgAN (Figures 4A,B).

**TABLE 1:** Overlapping DEGs among all selected four GEO datasets.

Human gene symbol	Gene full name	IgAN vs. HLD (GSE93798)		IgAN vs. HLD (GSE37460)		IgAN vs. HLD (GSE35487)		IgAN vs. HLD (GSE35488)	
		LogFC	Adjusted p-value	LogFC	Adjusted p-value	LogFC	Adjusted p-value	LogFC	Adjusted p-value
*ALB*	Albumin	−1.90	2.47E-08	−2.75	1.66E-04	−1.77	2.38E-02	−1.56	3.91E-02
*APOH*	Apolipoprotein	−1.21	2.88E-05	−1.85	1.71E-05	−1.06	2.18E-01	−1.13	2.06E-01
*ATF3*	Activating transcription factor 3	−2.09	2.45E-13	−2.40	1.85E-06	−2.70	6.36E-15	−2.72	8.13E-15
*BHLHE40*	Basic helix-loop-helix family member e40	−1.73	9.52E-12	−1.11	1.26E-05	−0.95	5.54E-05	−0.93	8.26E-06
*CRISPLD2*	Cysteine-rich secretory protein LCCL domain-containing 2	−1.23	8.62E-05	−1.36	9.38E-04	−0.62	6.87E-03	−0.59	5.33E-03
*CYP27B1*	Cytochrome P450 family 27 subfamily B member 1	−2.26	2.79E-14	−2.49	5.31E-07	−1.13	3.77E-03	−1.14	3.29E-03
*EGR1*	Early growth response 1	−2.75	1.98E-16	−1.65	6.95E-05	−2.32	2.50E-05	−1.49	2.36E-05
*EGR3*	Early growth response 3	−2.11	1.66E-05	−1.72	7.66E-04	−0.71	9.97E-07	−0.65	5.78E-07
*FOS*	Fos proto-oncogene, AP-1 transcription factor subunit	−4.63	1.03E-16	−1.40	1.80E-02	−1.85	2.16E-04	−1.89	9.58E-05
*FOSB*	FosB proto-oncogene, AP-1 transcription factor subunit	−6.29	7.74E-22	−3.53	8.06E-09	−2.48	1.23E-16	−2.51	7.57E-17
*G6PC*	Glucose-6-phosphatase catalytic subunit	−1.05	1.50E-07	−1.44	1.01E-03	−0.81	3.06E-01	−0.79	2.82E-01
*GADD45B*	Growth arrest and DNS damage inducible beta	−1.40	1.12E-09	−1.05	8.76E-05	−2.28	2.34E-08	−2.17	5.08E-08
*GDF15*	Growth differentiation factor 15	−1.86	6.12E-12	−1.67	3.01E-03	−1.86	6.52E-03	−1.89	3.53E-03
*GSTA1*	Glutathione S-transferase alpha	−2.14	2.51E-10	−1.78	5.88E-03	−0.60	2.60E-01	−0.63	2.60E-01
*HBB*	Hemoglobin subunit beta	1.03	2.12E-03	2.99	3.75E-05	1.96	1.07E-01	1.81	2.33E-01
*HBEGF*	Heparin-binding EGF-like growth factor	−1.21	1.51E-05	−1.12	1.51E-03	−1.57	7.99E-01	−1.01	1.68E-08
*HRG*	Histidine-rich glycoprotein	−1.30	4.92E-07	−1.12	7.92E-04	−0.76	2.12E-01	−0.61	1.81E-01
*MAFF*	MAF bZIP transcription factor F	−1.94	2.15E-08	−1.02	4.67E-03	−3.92	2.34E-08	−3.27	1.53E-10
*MYC*	MYC proto-oncogene	−1.56	1.88E-06	−1.03	1.39E-03	−0.81	1.07E-01	−0.80	6.72E-02
*NFIL3*	Nuclear factor, interleukin 3 regulated	−1.67	1.95E-11	−1.35	1.05E-05	−0.88	3.07E-03	−0.90	2.04E-03
*SLC19A2*	Solute carrier family 19 member 2	−1.64	2.92E-13	−1.02	5.62E-05	−0.72	1.00E-03	−0.73	4.23E-04

IgAN, IgA nephropathy; HLD, healthy living donor; LogFC, log2 fold change; GEO, Gene Expression Omnibus.

## Discussion

In the current study, we studied both glomerular and tubulointerstitial mRNA expression profiles by computational analysis and present novel observations of immune filtration involvement in the IgAN patients. Our current study revealed clear separation between the IgAN patients and healthy living controls by the distinct landscape of infiltrating immune cells, highlighting the role of infiltrating immune cells as one of the driving forces in the pathogenesis of IgAN. Specifically, our findings identified significantly increased proportions of M1 and M2 macrophages in the glomerular compartment as well as significantly high fractions of M2 macrophage and resting CD4^+^ memory T cells in the tubulointerstitial compartment from IgAN patients compared to control healthy subjects. Moreover, to explore the similarities and differences between glomerular and tubulointerstitial compartments, 21 DEGs were recognized as the commonly shared regulators between these two entities in the pathogenesis of IgAN, among which the *HBB* gene was positively associated with M2 macrophage infiltration in the glomerular compartment and resting CD4^+^ memory T cells infiltration in the tubulointerstitial compartment of IgAN patients. Most importantly, we confirmed the negative association of *FOSB* gene in the activation of M2 macrophage in the glomerular compartment of IgAN patients. Overall, our findings suggest a potentially important role of infiltrating immune cells, particularly M2 macrophages, in the pathophysiology of IgAN-associated renal damage, in which *FOSB* and *HBB* genes may provide important roles in this immune response.

The positive correlation between macrophages counts per glomerulus and the number of glomerular crescents in IgAN patients (Arima et al., 1991; Ikezumi et al., 2011; Silva et al., 2012; Xie et al., 2021) suggests an important role of the mononuclear phagocytes in the pathogenesis of mesangial hypercellularity and irreversible glomerular damage. Macrophages have been divided into classically activated M1 types that are characterized by a pro-inflammatory phenotype and alternatively activated M2 types that display regulatory functions in tissue repair and remodeling. In a study of 204 patients with IgAN, the presence of CD68^+^ (a marker of pan-macrophage or M1 type) macrophages was positively correlated with the following: Serum creatinine at the time of biopsy, proteinuria, progression of renal disease, and a worse disease outcome (Myllymaki et al., 2007; Soares et al., 2019). In line with this previous evidence, our current study demonstrates an obvious higher proportion of M1 macrophage in the glomerular compartment, strengthening the key role of M1 macrophage in the development of IgAN.

In the meantime, we observed increased M2 macrophage fractions in both the compartments of glomerular and tubulointerstitial, although there is still controversy regarding the long-term influence of M2 macrophage in the pathogenesis of IgAN. CD163^+^ (a marker of M2 type) M2 macrophages play a central role in hemoglobin clearance and limit oxidative heme toxicity, which are significant prognostic factors for an incomplete recovery of renal function in IgAN patients with macro-hematuria-associated acute kidney injury, warfarin coagulopathy, and paroxysmal nocturnal hemoglobinuria (Martin Cleary et al., 2010; Ballarin et al., 2011; Gutierrez et al., 2012). Despite the renoprotective role, emerging evidence suggests a distinct role of M2 macrophage to promote the development of IgAN. For example, the CD163^+^ M2 macrophages were found to be higher in both glomerular and tubulointerstitial compartments of kidney tissue from new-onset IgAN patients or IgAN patients with crescents (Ikezumi et al., 2010; Ikezumi et al., 2011; Silva et al., 2012; Han et al., 2013; Kim et al., 2015), and the glomerular M2 macrophage counts were positively correlated with percentage of crescents and fibrotic lesion (Ikezumi et al., 2011; Li et al., 2015; Zhao et al., 2015). Taken together, our observations, in accordance with previous evidence, precipitate the conclusion that M2 macrophages are positioned as potential effectors of glomerular and tubulointerstitial injury in IgAN and may be potential target for therapeutic intervention.

CD4^+^ T cells play critical in orchestrating B-cell responses (Yamada et al., 2010), and cytokines produced by subpopulations of CD4^+^ T cells can dramatically impact B-cell responses in IgAN pathogenesis and correlate with its clinical severity (Ruszkowski et al., 2019). Importantly, resting CD4^+^ memory T cells have been shown to be superior helpers for precipitating B-cell antibody production in comparison with naïve CD4^+^ T cells, and this is most readily derived from the capability of memory cells to more quickly attain a helper-competent activation state than naive cells (McKinstry et al., 2010). Our study agrees with these previous findings to show the apparently increased proportion of resting CD4^+^ memory T cells in the tubulointerstitial compartment of IgAN. Therefore, we presume that the unique functional qualities of resting CD4^+^ memory T cells have the strength to elicit a secondary response in diverse approaches *via* its continuum.

Further efforts are urgently needed in numerous areas if the clinically feasible imbalance of mononuclear phagocytes and T-cell subpopulations are highlighted as promising therapeutic targets and biomarkers for the treatment and monitoring of disease to succeed. Notably, the diagnostic role of mononuclear phagocytes and specific T cells have not been confirmed in studies of adequate sample size, different patient populations, and in patients with other forms of glomerulonephritis as controls (Yamada et al., 2010; Ruszkowski et al., 2019; Soares et al., 2019; Zheng et al., 2020). As a tool for risk stratification and disease monitoring, data on the association between mononuclear phagocytes, various T-cell subsets and the degree of histological damage, and the rate of decline in renal function as well as renal end points are limited. In addition, no data exist on the serial monitoring of these immune cell subpopulation changes at different phases of IgAN. Accordingly, future studies to validate their roles in risk stratification and monitoring of patients with IgAN, preferably in different stages of the disease and using several different populations of patients, are required before advancements can be made.

The KEGG enrichment analysis mainly reflect transcripts associated with some classic signaling pathways associated with immune cell activation and inflammation during the pathogenesis of IgAN, such as PPAR signaling pathway in the glomerular compartment but IL-17, tumor necrosis factor (TNF), and the Forkhead box O (FoxO) signaling pathways in the tubulointerstitial compartment. The PPAR signaling pathway has long been recognized as an important transcriptional activator in macrophage polarization toward the M2 phenotype (Chang et al., 2015), which to some extent is consistent with our current finding of significantly increased M2 macrophage in glomeruli. Similarly, IL-17, TNF, and FoxO signaling pathways are able to regulate the frequency of effector and/or memory CD4^+^ T cells by providing proliferative and survival signals either directly to the T cells or to the antigen-presenting cells with which they interact (Nembrini et al., 2009; Chen et al., 2016; Delpoux et al., 2021), which is in line with our current findings of apparently elevated resting CD4^+^ memory T cells in tubulointerstitial.

Few studies have pinpointed the commonly shared gene-expressing changes occurring in the glomerular and tubulointerstitial compartments using a sensitive transcriptomic profiling approach. Our study for the first time revealed that 21 DEGs were shared in both glomerular and interstitial compartments, including 1 upregulated gene (*HBB*) and 20 downregulated genes. These commonly shared DEGs indicated that these two different entities share some general mechanisms. However, in contrast to the positive association between *HBB* gene and CD8^+^ T-cell activation in the glomerular compartment, a negative association between *HBB* gene and CD8^+^ T-cell activation was observed in the tubulointerstitial entity. This discrepancy indicates that these two entities also differ from each other in the underlying pathophysiology. Our study further found that *HBB* gene was positively associated with M2 macrophage activation in the glomerular compartment and CD4^+^ memory T-cell activation in the tubulointerstitial compartment. The *HBB* gene provides instructions for generating a protein called beta-globin that is a subunit of a larger protein called hemoglobin. Although how the *HBB* gene expression in nonerythroid cells affected cellular physiology remains largely undetermined, the capability of hemoglobin in maintaining oxygen homeostasis and mediating inflammatory response in some other cell types precipitates one hypothesis that the binding of soluble hemoglobin to a yet unidentified hemoglobin receptor expressed in infiltrating immune cells (Saha et al., 2014). Such a receptor could facilitate the internalization of HBB into the target immune cells to elicit a series of subsequent immune response in IgAN. The elucidation of the mechanism underlying the activity of hemoglobin in exaggerating the immune response and boosting the development of IgAN has to be elucidated yet.

FBJ murine osteosarcoma viral oncogene homolog B (FOSB) is a protein that is encoded by the *FOSB* gene localized on chromosome 19q13 and composed of four exons in humans. *FOSB* gene is a member of the *Fos* family of genes that functions as transcription factors by forming the transcription factor complex active protein 1 (AP-1). AP-1 acts as a regulator of cell homeostasis and mediates gene expression in response to a variety of environmental and physiological factors such as cytokines, oxidative stress, and other cell stressors. It was confirmed in various experimental systems that renal inflammation, fibrosis, and podocyte function are deteriorated by elevating AP-1 complex composition (Mezzano et al., 2001; Le et al., 2005), indicating a potential regulatory effect of *FOSB* gene in the development of IgAN. Indeed, several clinical studies were consistent with our current study to identify the downregulated expression of *FOSB* in the kidney from IgAN patients, cultured podocytes, or tubule cells exposed to polymeric IgA extracted and purified from the serum of IgAN patients (Miraji et al., 2019; Zhang et al., 2019; Liao et al., 2020; Park et al., 2020). More importantly, *FOSB* gene overexpression in cultured podocyte or tubule cells was able to reduce the secretion of pro-inflammatory mediators (Liao et al., 2020). Special noteworthy is that our current study revealed not only an obvious deficiency of *FOSB* gene in the glomerular compartment of IgAN but also a close relation between the glomerular *FOSB* deficiency and M2 macrophage infiltration. It thus seems reasonable to speculate that the decreased *FOSB* gene expression in glomeruli might be tightly involved in the development of IgAN, possibly thorough a boosting influence on M2 macrophage activation (Ranghino et al., 2017).

Altogether, abnormal expression of effector molecules and disordered activation and differentiation of immune cells synergistically stimulates local inflammation and immune response, causing renal tissue damage and pathological repair in IgAN. Aiming at all aspects of immune mechanism involvement, it is helpful to develop novel and promising clinical early-diagnosis and prognostic indicators as well as ideal specific therapeutic targets in translational medicine. Notably, in the current study, the identification of mononuclear phagocytes and specific T cells involved in the occurrence and development of IgAN highlights the imbalance of these immune cell subsets as new therapeutic targets and biomarkers for the treatment and monitoring of the disease.

## Conclusion

To the best of our knowledge, no study has analyzed the immune cell transcriptome within the renal microenvironments of IgAN and associated molecular patterns through a combination of all the currently available datasets; the original studies derived from all the selected datasets (Reich et al., 2010; Berthier et al., 2012; Liu et al., 2017) have not performed any microarray analysis regarding the immune cell states, either. Therefore, we employed a cross-dataset integration strategy of defining the landscape of the immune cells and associated molecular patterns from human renal biopsies of IgAN. Our current study for the first time revealed that the diversity in the immune cell landscape could clearly separate IgAN patients from healthy living controls, and enhanced M2 macrophage infiltration possibly plays an important role in boosting the pathogenesis of IgAN in both glomerular and tubulointerstitial compartments. In specific, glomerular elevation of *HBB* gene and deficiency of *FOSB* gene were found to be closely associated with M2 macrophage infiltration, suggesting the potential role of these two DEGs in keeping the differentiation toward a M2-skewed polarization phenotype, which consequently triggers the immune response and the renal damage of IgAN. These findings may be useful to develop M2 macrophage-targeted therapy for IgAN treatment in the future.

## Data Availability

The original contributions presented in the study are included in the article/Supplementary Material, further inquiries can be directed to the corresponding author.

## References

[B1] Arima S. Nakayama M. Naito M. Sato T. Takahashi K. (1991). Significance of Mononuclear Phagocytes in IgA Nephropathy. Kidney Int. 39, 684–692. 10.1038/ki.1991.82 2051725

[B2] Ballarin J. Arce Y. Torra Balcells R. Diaz Encarnacion M. Manzarbeitia F. Ortiz A. (2011). Acute Renal Failure Associated to Paroxysmal Nocturnal Haemoglobinuria Leads to Intratubular Haemosiderin Accumulation and CD163 Expression. Nephrol. Dial. Transplant. 26, 3408–3411. 10.1093/ndt/gfr391 21771756

[B3] Berthier C. C. Bethunaickan R. Gonzalez-Rivera T. Nair V. Ramanujam M. Zhang W. (2012). Cross-species Transcriptional Network Analysis Defines Shared Inflammatory Responses in Murine and Human Lupus Nephritis. J. Immunol. 189, 988–1001. 10.4049/jimmunol.1103031 22723521 PMC3392438

[B4] Chang H. Y. Lee H.-N. Kim W. Surh Y.-J. (2015). Docosahexaenoic Acid Induces M2 Macrophage Polarization through Peroxisome Proliferator-Activated Receptor γ Activation. Life Sci. 120, 39–47. 10.1016/j.lfs.2014.10.014 25445227

[B5] Chen X. Nie Y. Xiao H. Bian Z. Scarzello A. J. Song N.-Y. (2016). TNFR2 Expression by CD4 Effector T Cells Is Required to Induce Full-Fledged Experimental Colitis. Sci. Rep. 6, 32834. 10.1038/srep32834 27601345 PMC5013387

[B6] Delpoux A. Marcel N. Hess Michelini R. Katayama C. D. Allison K. A. Glass C. K. (2021). FOXO1 Constrains Activation and Regulates Senescence in CD8 T Cells. Cel Rep. 34, 108674. 10.1016/j.celrep.2020.108674 33503413

[B7] Gutierrez E. Egido J. Rubio-Navarro A. Buendía I. Blanco Colio L. M. Toldos O. (2012). Oxidative Stress, Macrophage Infiltration and CD163 Expression Are Determinants of Long-Term Renal Outcome in Macrohematuria-Induced Acute Kidney Injury of IgA Nephropathy. Nephron Clin. Pract. 121, c42–c53. 10.1159/000342385 23095372

[B8] Han Y. Ma F. Y. Tesch G. H. Manthey C. L. Nikolic-Paterson D. J. (2013). Role of Macrophages in the Fibrotic Phase of Rat Crescentic Glomerulonephritis. Am. J. Physiol. Renal Physiol. 304, F1043–F1053. 10.1152/ajprenal.00389.2012 23408165

[B9] Ikezumi Y. Suzuki T. Karasawa T. Hasegawa H. Kawachi H. Nikolic-Paterson D. J. (2010). Contrasting Effects of Steroids and Mizoribine on Macrophage Activation and Glomerular Lesions in Rat Thy-1 Mesangial Proliferative Glomerulonephritis. Am. J. Nephrol. 31, 273–282. 10.1159/000279163 20110667

[B10] Ikezumi Y. Suzuki T. Karasawa T. Hasegawa H. Yamada T. Imai N. (2011). Identification of Alternatively Activated Macrophages in New-Onset Paediatric and Adult Immunoglobulin A Nephropathy: Potential Role in Mesangial Matrix Expansion. Histopathology 58, 198–210. 10.1111/j.1365-2559.2011.03742.x 21323947

[B11] Kim M.-G. Kim S. C. Ko Y. S. Lee H. Y. Jo S.-K. Cho W. (2015). The Role of M2 Macrophages in the Progression of Chronic Kidney Disease Following Acute Kidney Injury. PloS one 10, e0143961. 10.1371/journal.pone.0143961 26630505 PMC4667939

[B12] Le N. H. van der Wal A. van der Bent P. Lantinga-van Leeuwen I. S. Breuning M. H. van Dam H. (2005). Increased Activity of Activator Protein-1 Transcription Factor Components ATF2, C-Jun, and C-Fos in Human and Mouse Autosomal Dominant Polycystic Kidney Disease. J. Am. Soc. Nephrol. 16, 2724–2731. 10.1681/asn.2004110913 16049073

[B13] Li J. Liu C. H. Gao B. Xu D. L. (2015). Clinical-pathologic Significance of CD163 Positive Macrophage in IgA Nephropathy Patients with Crescents. Int. J. Clin. Exp. Med. 8, 9299–9305. 26309588 PMC4538096

[B14] Liao Y. Wang Z. Wang L. Lin Y. Ye Z. Zeng X. (2020). MicroRNA-27a-3p Directly Targets FosB to Regulate Cell Proliferation, Apoptosis, and Inflammation Responses in Immunoglobulin a Nephropathy. Biochem. Biophys. Res. Commun. 529, 1124–1130. 10.1016/j.bbrc.2020.06.115 32819575

[B15] Liu P. Lassén E. Nair V. Berthier C. C. Suguro M. Sihlbom C. (2017). Transcriptomic and Proteomic Profiling Provides Insight into Mesangial Cell Function in IgA Nephropathy. J. Am. Soc. Nephrol. 28, 2961–2972. 10.1681/asn.2016101103 28646076 PMC5619958

[B16] Martin Cleary C. Moreno J. A. Fernandez B. Ortiz A. Parra E. G. Gracia C. (2010). Glomerular Haematuria, Renal Interstitial Haemorrhage and Acute Kidney Injury. Nephrol. Dial. Transplant. 25, 4103–4106. 10.1093/ndt/gfq493 20709744

[B17] McKinstry K. K. Strutt T. M. Swain S. L. (2010). The Potential of CD4 T-Cell Memory. Immunology 130, 1–9. 10.1111/j.1365-2567.2010.03259.x 20331470 PMC2855787

[B18] Mezzano S. A. Barría M. Droguett M. A. Burgos M. E. Ardiles L. G. Flores C. (2001). Tubular NF-κB and AP-1 Activation in Human Proteinuric Renal Disease. Kidney Int. 60, 1366–1377. 10.1046/j.1523-1755.2001.00941.x 11576350

[B19] Miraji M. K. Cheng Y. Ge S. Xu G. (2019). Identification of Primary Genes in Glomeruli Compartment of Immunoglobulin A Nephropathy by Bioinformatic Analysis. PeerJ 7, e7067. 10.7717/peerj.7067 31355054 PMC6645034

[B20] Myllymaki J. M. Honkanen T. T. Syrjänen J. T. Helin H. J. Rantala I. S. Pasternack A. I. (2007). Severity of Tubulointerstitial Inflammation and Prognosis in Immunoglobulin A Nephropathy. Kidney Int. 71, 343–348. 10.1038/sj.ki.5002046 17191083

[B21] Nembrini C. Marsland B. J. Kopf M. (2009). IL-17-producing T Cells in Lung Immunity and Inflammation. J. Allergy Clin. Immunol. 123, 986–994. 10.1016/j.jaci.2009.03.033 19410688

[B22] Park S. Yang S. H. Jeong C. W. Moon K. C. Kim D. K. Joo K. W. (2020). RNA-seq Profiling of Microdissected Glomeruli Identifies Potential Biomarkers for Human IgA Nephropathy. Am. J. Physiol. Renal Physiol. 319, F809–F821. 10.1152/ajprenal.00037.2020 32954852

[B23] Ranghino A. Bruno S. Bussolati B. Moggio A. Dimuccio V. Tapparo M. (2017). The Effects of Glomerular and Tubular Renal Progenitors and Derived Extracellular Vesicles on Recovery from Acute Kidney Injury. Stem Cel Res. Ther. 8, 24. 10.1186/s13287-017-0478-5 PMC5297206 28173878

[B24] Reich H. N. Tritchler D. Cattran D. C. Herzenberg A. M. Eichinger F. Boucherot A. (2010). A Molecular Signature of Proteinuria in Glomerulonephritis. PloS one 5, e13451. 10.1371/journal.pone.0013451 20976140 PMC2956647

[B25] Ruszkowski J. Lisowska K. A. Pindel M. Heleniak Z. Dębska-Ślizień A. Witkowski J. M. (2019). T Cells in IgA Nephropathy: Role in Pathogenesis, Clinical Significance and Potential Therapeutic Target. Clin. Exp. Nephrol. 23, 291–303. 10.1007/s10157-018-1665-0 30406499 PMC6394565

[B26] Saha D. Patgaonkar M. Shroff A. Ayyar K. Bashir T. Reddy K. V. (2014). Hemoglobin Expression in Nonerythroid Cells: Novel or Ubiquitous? Int. J. Inflam. 2014, 803237. 10.1155/2014/803237 25431740 PMC4241286

[B27] Silva G. Costa R. Ravinal R. Ramalho L. Reis M. Moyses-Neto M. (2012). Renal Macrophage Infiltration Is Associated with a Poor Outcome in IgA Nephropathy. Clinics 67, 697–703. 10.6061/clinics/2012(07)01 22892911 PMC3400157

[B28] Soares M. F. Genitsch V. Chakera A. Smith A. MacEwen C. Bellur S. S. (2019). Relationship between Renal CD68+ Infiltrates and the Oxford Classification of IgA Nephropathy. Histopathology 74, 629–637. 10.1111/his.13768 30303541

[B29] Xie D. Zhao H. Xu X. Zhou Z. Su C. Jia N. (2021). Intensity of Macrophage Infiltration in Glomeruli Predicts Response to Immunosuppression in Patients with IgA Nephropathy. J. Am. Soc. Nephrol. 32, 3187. 10.1681/ASN.2021060815 PMC8638408 34670812

[B30] Yamada K. Kobayashi N. Ikeda T. Suzuki Y. Tsuge T. Horikoshi S. (2010). Down-regulation of Core 1 1,3-galactosyltransferase and Cosmc by Th2 Cytokine Alters O-Glycosylation of IgA1. Nephrol. Dial. Transplant. 25, 3890–3897. 10.1093/ndt/gfq325 20551088 PMC2989791

[B31] Zhang D. Cao Y. Zuo Y. Wang Z. Mi X. Tang W. (2019). Integrated Bioinformatics Analysis Reveals Novel Hub Genes Closely Associated with Pathological Mechanisms of Immunoglobulin A Nephropathy. Exp. Ther. Med. 18, 1235–1245. 10.3892/etm.2019.7686 31316619 PMC6601137

[B32] Zhao L. David M. Z. Hyjek E. Chang A. Meehan S. M. (2015). M2 Macrophage Infiltrates in the Early Stages of ANCA-Associated Pauci-Immune Necrotizing GN. Clin. J. Am. Soc. Nephrol. 10, 54–62. 10.2215/cjn.03230314 25516918 PMC4284408

[B33] Zheng Y. Lu P. Deng Y. Wen L. Wang Y. Ma X. (2020). Single-Cell Transcriptomics Reveal Immune Mechanisms of the Onset and Progression of IgA Nephropathy. Cel Rep. 33, 108525. 10.1016/j.celrep.2020.108525 33357427

