# Supplementary material for: Computational Analysis Reveals the Characteristics of Immune Cells in Glomerular and Tubulointerstitial Compartments in IgA Nephropathy Patients
Source: Front Genet. 2022 May 4;13:838863. doi: 10.3389/fgene.2022.838863 (PMC9116531; doi:10.3389/fgene.2022.838863)
Supplement: Supplementary file 2 [file Table1.DOCX]

**Table 1∣Overlapping DEGs among all selected 4 GEO datasets**

|  |  | **IgAN vs. HLD (GSE93798)** | | **IgAN vs. HLD (GSE37460)** | | **IgAN vs. HLD (GSE35487)** | | **IgAN vs. HLD (GSE35488)** | |
| --- | --- | --- | --- | --- | --- | --- | --- | --- | --- |
| **Human Gene Symbol** | **Gene full name** | **LogFC** | **Adjusted p-value** | **LogFC** | **Adjusted p-value** | **LogFC** | **Adjusted p-value** | **LogFC** | **Adjusted p-value** |
| *ALB* | Albumin | -1.90 | 2.47E-08 | -2.75 | 1.66E-04 | -1.77 | 2.38E-02 | -1.56 | 3.91E-02 |
| *APOH* | Apolipoprotein | -1.21 | 2.88E-05 | -1.85 | 1.71E-05 | -1.06 | 2.18E-01 | -1.13 | 2.06E-01 |
| *ATF3* | Activating transcription factor 3 | -2.09 | 2.45E-13 | -2.40 | 1.85E-06 | -2.70 | 6.36E-15 | -2.72 | 8.13E-15 |
| *BHLHE40* | Basic helix-loop-helix family member e40 | -1.73 | 9.52E-12 | -1.11 | 1.26E-05 | -0.95 | 5.54E-05 | -0.93 | 8.26E-06 |
| *CRISPLD2* | Cysteine rich secretory protein LCCL domain containing 2 | -1.23 | 8.62E-05 | -1.36 | 9.38E-04 | -0.62 | 6.87E-03 | -0.59 | 5.33E-03 |
| *CYP27B1* | Cytochrome P450 family 27 subfamily B member 1 | -2.26 | 2.79E-14 | -2.49 | 5.31E-07 | -1.13 | 3.77E-03 | -1.14 | 3.29E-03 |
| *EGR1* | Early growth response 1 | -2.75 | 1.98E-16 | -1.65 | 6.95E-05 | -2.32 | 2.50E-05 | -1.49 | 2.36E-05 |
| *EGR3* | Early growth response 3 | -2.11 | 1.66E-05 | -1.72 | 7.66E-04 | -0.71 | 9.97E-07 | -0.65 | 5.78E-07 |
| *FOS* | Fos proto-oncogene, AP-1 transcription factor subunit | -4.63 | 1.03E-16 | -1.40 | 1.80E-02 | -1.85 | 2.16E-04 | -1.89 | 9.58E-05 |
| *FOSB* | FosB proto-oncogene, AP-1 transcription factor subunit | -6.29 | 7.74E-22 | -3.53 | 8.06E-09 | -2.48 | 1.23E-16 | -2.51 | 7.57E-17 |
| *G6PC* | Glucose-6-phosphatase catalytic subunit | -1.05 | 1.50E-07 | -1.44 | 1.01E-03 | -0.81 | 3.06E-01 | -0.79 | 2.82E-01 |
| *GADD45B* | Growth arrest and DNS damage inducible beta | -1.40 | 1.12E-09 | -1.05 | 8.76E-05 | -2.28 | 2.34E-08 | -2.17 | 5.08E-08 |
| *GDF15* | Growth differentiation factor 15 | -1.86 | 6.12E-12 | -1.67 | 3.01E-03 | -1.86 | 6.52E-03 | -1.89 | 3.53E-03 |
| *GSTA1* | Glutathione S-transferase alpha | -2.14 | 2.51E-10 | -1.78 | 5.88E-03 | -0.60 | 2.60E-01 | -0.63 | 2.60E-01 |
| *HBB* | Hemoglobin subunit beta | 1.03 | 2.12E-03 | 2.99 | 3.75E-05 | 1.96 | 1.07E-01 | 1.81 | 2.33E-01 |
| *HBEGF* | Heparin binding EGF like growth factor | -1.21 | 1.51E-05 | -1.12 | 1.51E-03 | -1.57 | 7.99E-01 | -1.01 | 1.68E-08 |
| *HRG* | Histidine rich glycoprotein | -1.30 | 4.92E-07 | -1.12 | 7.92E-04 | -0.76 | 2.12E-01 | -0.61 | 1.81E-01 |
| *MAFF* | MAF bZIP transcription factor F | -1.94 | 2.15E-08 | -1.02 | 4.67E-03 | -3.92 | 2.34E-08 | -3.27 | 1.53E-10 |
| *MYC* | MYC proto-oncogene | -1.56 | 1.88E-06 | -1.03 | 1.39E-03 | -0.81 | 1.07E-01 | -0.80 | 6.72E-02 |
| *NFIL3* | Nuclear factor, interleukin 3 regulated | -1.67 | 1.95E-11 | -1.35 | 1.05E-05 | -0.88 | 3.07E-03 | -0.90 | 2.04E-03 |
| *SLC19A2* | Solute carrier family 19 member 2 | -1.64 | 2.92E-13 | -1.02 | 5.62E-05 | -0.72 | 1.00E-03 | -0.73 | 4.23E-04 |

IgAN, IgA nephropathy; HLD, healthy living donor; LogFC, log2 ^fold change^, GEO, gene expression omnibus.
