# Supplementary material for: Computational Analysis Reveals the Characteristics of Immune Cells in Glomerular and Tubulointerstitial Compartments in IgA Nephropathy Patients
Source: Front Genet. 2022 May 4;13:838863. doi: 10.3389/fgene.2022.838863 (PMC9116531; doi:10.3389/fgene.2022.838863)
Supplement: Supplementary file 4 [file Table2.DOCX]

**Supplemental Table S1∣Profiling datasets of Gene Expression Omnibus (GEO).**

| **GEO ID** | **Species** | **Renal compartment** | **Overall Design** | **Groups Definition** | **Platform** | **Year** | **Country** | **Reference** |
| --- | --- | --- | --- | --- | --- | --- | --- | --- |
| GSE93798 | Human | Glomeruli | RNA from glomeruli was extracted and processed for hybridization on Affymetrix microarrays. | (1) Control human kidney (n=22, GSM2462533-GSM2462554)  (2) IgA Nephropathy (n=20, GSM2462555-GSM2462574) | GPL22945, [HG-U133_Plus_2] Affymetrix Human Genome U133 Plus 2.0 Array [CDF: Brainarray HGU133Plus2_Hs_ENTREZG_v19] | 2017 | USA | (Liu et al., 2017) |
| GSE37460 | Human | Glomeruli | RNA from glomeruli was extracted and processed for hybridization on Affymetrix microarrays. | (1) Healthy living donor (n=27, GSM920350-GSM920353, GSM920368-GSM920385, GSM1046922-GSM1046926)  (2) IgA Nephropathy (n=27, GSM920401-GSM920427) | GPL11670, Affymetrix Human Genome U133 Plus 2.0 Array [Hs133P_Hs_ENTREZG.cdf]  GPL14663, Affymetrix GeneChip Human Genome HG-U133A Custom CDF [Affy_HGU133A_CDF_ENTREZG_10] | 2012 | USA | (Berthier et al., 2012) |
| GSE35487 | Human | Tubulointerstitial | RNA from tubulointerstitial was extracted and processed for hybridization on Affymetrix microarrays. | (1) Control living donor (n=6, GSM869339-GSM869344)  (2) IgA Nephropathy (n=28, GSM869311-GSM869338) | GPL96, [HG-U133A] Affymetrix Human Genome U133A Array | 2012 | USA | (Reich et al., 2010) |
| GSE35488 | Human | Tubulointerstitial | RNA from tubulointerstitial was extracted and processed for hybridization on Affymetrix microarrays. | (1) Control living donor (n=6, GSM869370-GSM869375)  (2) IgA Nephropathy (n=25, GSM869345-GSM869369) | GPL14663, Affymetrix GeneChip Human Genome HG-U133A Custom CDF [Affy_HGU133A_CDF_ENTREZG_10] | 2012 | USA | (Reich et al., 2010) |

References:

Berthier, C.C., Bethunaickan, R., Gonzalez-Rivera, T., Nair, V., Ramanujam, M., Zhang, W., Bottinger, E.P., Segerer, S., Lindenmeyer, M., Cohen, C.D.*, et al.* (2012). Cross-species transcriptional network analysis defines shared inflammatory responses in murine and human lupus nephritis. J Immunol *189*, 988-1001.

Liu, P., Lassen, E., Nair, V., Berthier, C.C., Suguro, M., Sihlbom, C., Kretzler, M., Betsholtz, C., Haraldsson, B., Ju, W.*, et al.* (2017). Transcriptomic and Proteomic Profiling Provides Insight into Mesangial Cell Function in IgA Nephropathy. J Am Soc Nephrol *28*, 2961-2972.

Reich, H.N., Tritchler, D., Cattran, D.C., Herzenberg, A.M., Eichinger, F., Boucherot, A., Henger, A., Berthier, C.C., Nair, V., Cohen, C.D.*, et al.* (2010). A molecular signature of proteinuria in glomerulonephritis. PloS one *5*, e13451.
